# The Relationship between Personality Profiles and the Esthetic Perception of Orthodontic Appliances

**DOI:** 10.1155/2024/8827652

**Published:** 2024-04-23

**Authors:** Selma Pascoal, Maria Gonçalves, Pietro Salvador, Rui Azevedo, Manuela Leite, Teresa Pinho

**Affiliations:** ^1^UNIPRO—Oral Pathology and Rehabilitation Research Unit, University Institute of Health Sciences IUCS-CESPU, Gandra 4585-116, Portugal; ^2^Associate Laboratory i4HB—Institute for Health and Bioeconomy, University Institute of Health Sciences—CESPU, Gandra 4585-116, Portugal; ^3^UCIBIO—Applied Molecular Biosciences Unit, Translational Toxicology Research Laboratory, University Institute of Health Sciences (1H-TOXRUN, IUCS—CESPU), Gandra 4585-116, Portugal; ^4^UCIBIO—Applied Molecular Biosciences Unit, Forensics and Biomedical Sciences Research Laboratory, University Institute of Health Sciences (1H-TOXRUN, IUCS—CESPU), Gandra 4585-116, Portugal; ^5^iHealth4Well-being—Innovation in Health and Well-Being—Research Unit, Polytechnic University of Health, CESPU, CRL 4560-462 Penafiel, Gandra, Portugal

## Abstract

**Introduction:**

In orthodontics, patients' esthetic expectations involve the final esthetic result as well as the treatment's appliance choice. Personality traits can influence patients' perception, treatment modality selection, expectations, compliance, and satisfaction with the treatment outcome, although there are very few studies on this interconnection between personality and orthodontic appliances.

**Objective:**

The aim of this study is to evaluate the relationship between personality traits and the esthetic perception of different orthodontic appliances.

**Materials and Methods:**

The online questionnaire is composed of three parts: (1) sociodemographic variables; (2) questions on the esthetic perception of different orthodontic appliances; (3) general personality assessment via the NEO-FFI. A total of 461 questionnaires were accepted.

**Results:**

There were statistically significant differences between laypeople and professionals related to assessing smiles and the need for orthodontic treatment. Laypeople identified a statistically significant relationship between personality traits and orthodontic appliances, in which esthetic perception is associated with high traits of agreeableness and openness. Neuroticism is most associated with orthodontic treatment with fixed appliances, which are the most conventional.

**Conclusions:**

Professionals tend to have a more critical judgment than laypeople as far as esthetics is concerned. Personality traits play an important role in esthetic perception and may influence orthodontic treatment.

## 1. Introduction

Functional occlusion and esthetic smiles are the primary goals of modern dentistry [1–3]. The human face is often the feature that people observe first. As such, it plays a fundamental role in the development of an individual's self-esteem and self-image, as positive social interactions have been shown to result in better interpersonal relationships and more self-confidence [4].

Dental esthetics are becoming increasingly important: the dental field has seen a particular increase in attention to orthodontic care due to the dominant role played by smiles and perioral areas in people's lives [5–7].

Patients' esthetic expectations are not only related to the final esthetic result but also to the treatment's appliance choice. The concern for highly visible orthodontic appliances has also prompted the development of more esthetic solutions, such as the lingual technique, composite and ceramic material brackets, esthetic archwire, up to clear aligners [8].

Today, almost every orthodontic treatment can have multiple approaches, and patients considering treatment can choose from many available appliances. Considering patients' esthetic self-perception, practitioners define a unique treatment plan and choose the best appliances in order to get their compliance [9].

Patients' approval, perception, and satisfaction with oral health and treatment might not be secured even if they receive adequate dental treatment. Personality attributes might underlie and explain such observations [10–12].

Personality traits are relatively stable over time because of a broad influence of heritability in the range of 40%–55%, and sex differences in hereditability are not large since it appears that the same genes operate on all traits in both sexes [13]. The NEO Five-Factor Inventory (NEO-FFi) includes the following traits: neuroticism, extraversion, openness, agreeableness, and conscientiousness. Extraversion and neuroticism are the strongest predictors of subjective well-being, and agreeableness and conscientiousness, to some extent, predispose individuals toward well-being. It allows a complete assessment of personality characteristics, and it is sensitive, highly valid, reliable, and accurate in measuring personality traits [14]. Also, it is easy to answer and score, interpret, and well documented in the literature [15].

Some studies have highlighted personality characteristics as intrinsic factors that affect patients' motivation for orthodontic treatment. Some authors have reported that psychological disorders can lead the patient to miss orthodontic appointments; however, others have reported that personality traits do not, by themselves, predict cooperation during treatment [5–7].

The different perceptions of the esthetic and functional priorities of dentists and patients can lead patients away from accepting proposed treatment plans. It is therefore important, on the one hand, to identify the most relevant parameters that allow increasing the satisfaction of patients undergoing treatments and, on the other hand, that promote receptiveness of the patients, namely convincing those with higher esthetic standards and in more advanced age groups, who are usually more distant from this type of treatment option [16–18].

To date, few studies have investigated the effect of orthodontic treatment on personality traits and individuals' general perception of different orthodontic appliances, so it is worth of investigating these aspects in an attempt to provide more evidence of the options available and their impacts.

In this context, the aim of this study is to evaluate the relationship between personality traits and the esthetic perception of different orthodontic appliances. The specific aims are (1) to compare smile evaluation and choice of orthodontic treatment between laypeople and dentistry workers and (2) to evaluate the relationship between personality traits, smile evaluation, and orthodontic appliance choice in the laypeople group.

The hypotheses to be tested in the population included in this study were as follows:

- H0: The smile evaluation and choice of orthodontic treatment do not differ significantly between laypeople and dentistry workers.
- H0: The smile evaluation does not differ significantly between the different personality traits in laypeople.
- H0: The orthodontic appliance choice does not differ significantly between the different personality traits.

## 2. Materials and Methods

### 2.1. Study Design

The present study was performed in ethical harmony with the Helsinki Declaration (9th version, 2013), following a within-subject, cross-sectional observational design. It was authorized by the Ethics Committee of the Instituto Universitário de Ciências da Saúde (reference number CE/IUCS/CESPU-11/21). Participants were provided with a thorough explanation of different aspects of this investigation. Informed consent was obtained from all participants for the experiment. They also had the option to withdraw from the study at any time through nonsubmission of the final questionnaire, which would be automatically excluded.

It was a convenience sample, collected through the “Snowball” method, in which the questionnaire (Questionnaire S1) carried out in Lime Survey 5.0.1 was shared through social networks and personalized contacts to university students, dentists, and other individuals (messages via WhatsApp, Messenger, Instagram, and e-mail), informing about the purpose of the study, inviting them to participate and share it with their contacts. The only inclusion criteria was to be 18 years or older.

The participants with no experience in dentistry were regarded as “Laypeople,” while individuals like dentists, dental hygienists, prosthetic technicians, and assistants were classified as “Dentistry Workers.”

### 2.2. Sample

A total of 461 questionnaires were fully completed and accepted for inclusion in the study, with a predominance of the female gender, accounting for 79.8%. The ages range from 18 to 70, with the majority in the 18–30 age group. With regard to academic qualifications, most of the sample of participants have a Master's degree or Bachelor's; of the remainder, almost half have an A level or less. The distribution between Laypeople and Dentistry workers (such as dentists, oral hygienists, prosthetic technicians, and assistants) is also presented, with most of the sample dominated by laypeople (69.8%).

### 2.3. Tool and Procedures

**Figure 1:**
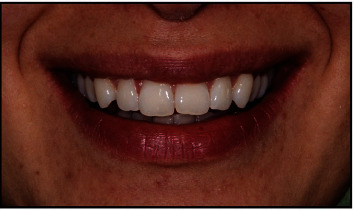
The model's smile.

**Figure 2:**
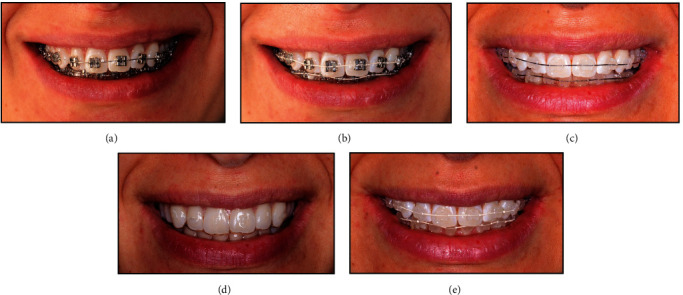
(a) Metallic Brackets RMO trimorphic (Rocky Mountain Orthodontics®) with Metallic Wire Ni-Ti .012 (Leone®) (MB-MW). (b) Metallic Brackets RMO trimorphic (Rocky Mountain Orthodontics®) with Aesthetic Wire Ni-Ti aesthetic Full Form (Elude®) (MB-AW). (c) Composite Aesthetic Brackets DB OrthoFlex Roth (OrthoTechnology®) with Metallic Wire (AB-MW). (d) Polyurethane vacuum-formed Aligner with anterior attachments (AL). (e) Composite Aesthetic Brackets DB OrthoFlex Roth (OrthoTechnology®) with Aesthetic Wire (AB-AW).

An online questionnaire (Questionnaire S1) was used for sample collection, composed of (1) sociodemographic variables, (2) questions on the esthetic perception of different orthodontic appliances, and (3) NEO-FFI. Variables (1) and (2) were developed by the researchers. For each section, the following was recorded:

1. Sociodemographic variables: gender, age, ethnicity, education, dental education, and orthodontic treatment history.
2. Esthetic perception: Perception of orthodontic needs and general orthodontics appliance preferences: a group of questions concerning an esthetic rating of the natural smile of one model (Figure 1), perception of orthodontic needs, and general appliance preferences (Figure 2).

The images, incorporated into the questionnaire, represent the most popular devices used on orthodontic treatments and were taken from the same live model.

For the images depicting brackets, with the consent of the model, it was performed an atraumatic protocol using the white liquid dam Opal–Dam (Ultradent®), following “debonding” with Hu–Friedy college tweezer, brush, and toothpaste.

For the images depicting aligners, the attachments were placed on the aligners, filled with shade A2 G-Aenial Anterior (GC Europe®), and worn by the model. Attachments were not bonded to the model.

Images were taken with a Nikon D1000 Camera with AF-S Micro Nikkor 85 mm lens (Nikon Corporation) by a single photographer in the same location to ensure analogous lighting conditions and positioning of each photograph using a Flesh Metz Megabits 15MS-1 with fixed focus to 50 mm.

To minimize any distraction variables, the images were framed to display only the smile, excluding any other facial structures.

(3) NEO-FFI inventory: This inventory was developed by Costa and McCrae and has been translated and adapted in Portugal by de Lima et al. [14]. The NEO-FFI inventory has 60 questions that are scored on a 5-point Likert scale (response from strongly agree to strongly disagree).

### 2.4. Statistical Analysis

Data analysis was carried out using IBM® SPSS® Statistics v29.0 for Windows. The association between the groups and the categorical variables of interest was performed using Chi-square and Cramer's Phi tests. The Shapiro–Wilk test was used to assess sample normality, with no evidence for rejecting the null hypotheses. The normality of the data led us to adopt Student's *t*-test to compare the personality dimensions according to the type of orthodontic treatment chosen (fixed appliance or aligners) in the Laypeople group. To measure the magnitude of the effect, Cohen's *d* was used with the following guidelines: | *d* | ≤ 0.20 expected as a small effect, | *d* | = 0.50 as a moderate effect, and | *d* | ≥ 0.80 as a large effect [19]. To compare the personality traits according to the clinical characteristics of the sample an ANOVA test was applied. Effect sizes for ANOVA were determined using *η*^2^ values, considering the thresholds *η*^2^ = 0.01 for a small effect, *η*^2^ = 0.06 for a medium effect, and *η*^2^ = 0.14 for a large effect. The significance level was set at 0.05.

## 3. Results

### 3.1. Demographic Characteristics of the Groups

The demographic characteristics of the population studied are shown in Table 1. We observed a statistically significant relation between age and groups (*χ*^2^(4) = 23.52; *p* < 0.001), with 57.1% with the majority of laypeople aged between 18 and 31 years (57.1%), followed by the 40–49 years group (19.6%). The majority of dentistry workers are also aged 18–31 (36.7%), followed by 30–39 (22.3%).

### 3.2. Smile Evaluation

Upon presentation of the photo (Figure 1), the participants were asked to evaluate the smile. A total of 202 (62.7%) laypeople and 64 (46.0%) dentistry workers found the smile beautiful. Conversely, 12 (8.6%) dentistry workers and 2 (0.6%) laypeople considered it an ugly smile. A statistically significant relationship was found to exist (*χ*^2^(3) = 38.33; *p* < 0.001) (Table 2).

They were also asked, “If this were your smile, would you improve it with an orthodontic treatment?”. In this case, statistically significant differences are also found between the opinions of laypeople and dentistry workers (*χ*^2^(2) = 31.76; *p* < 0.001); more laypeople (179/55.6%) say no, and more dentistry workers (60/43.2%) say yes (Table 2).

### 3.3. Gender Comparison in Smile Rating

When we compare the two genders in how they evaluate the smile (Table 3), we find that in the laypeople group, most of the males (34/54%) and females (168/64.9%) consider the smile to be beautiful, and only one individual of each gender considers the smile to be ugly. In the dentistry workers group, a similar result, with most individuals of both genders considering the smile to be beautiful, although with 11 (10.1%) of the females and 1 (3.3%) of the males considering the smile to be ugly, a difference not statistically significant.

Regarding the question “If this were your smile, would you improve it with an orthodontic treatment?” in the laypeople group, the majority of males (66.7%) and females (52.9%) answered “No.” In the dentistry workers group, there is a divergence between male and female subjects, with the majority of males (15/50.0%) not improving their smile and the majority of females (50/45.9%) improving their smile.

### 3.4. Images Filtered by the Preference of Laypeople and Dentistry Workers

Participants were asked to rank the pictures in Figure 2. encompassing different types of orthodontic treatments according to their preference, placing their most favorite at the top and their least favorite at the bottom. The most voted image for each position is congruent between laypeople and dentistry workers. Almost 70% of both laypeople and dentistry workers chose the aligners as their number one preference (Figure 2(d)), followed by Figure 2(e) (AB–AW) for the second position, with approximately 40% of the votes; Figure 2(c) (AB–MW); Figure 2(b) (MB–AW); and in the fifth position, Figure 2(a) (MB–MW) (Tables 4 and 5).

### 3.5. Laypeople Analysis

- Differences in the personality traits of the laypeople group, according to the smile evaluation in Figure 1.

Statistically significant differences were found between the smile evaluation in relation to the personality dimensions: extraversion (*F* (3, 318) = 4.58; *p* = 0.003; *ɳ*^2^ = 0.044), openness (*F* (3, 318) = 3.9; *p* = 0.009; *ɳ*^2^ = 0.035), and agreeableness (*F* (3, 318) = 3.03; *p* = 0.03; *ɳ*^2^ = 0.028).

Participants who considered the smile to be very beautiful showed significantly higher values in the extraversion, openness, and agreeableness dimensions compared to the other evaluations (beautiful, neutral, and ugly), as shown in Table 6.

(b) Differences in the personality traits of the laypeople group, according to the question, “If this were your smile, would you improve it with an orthodontic treatment?”

By relating the personality dimensions to the question, “If this were your smile, would you improve it with an orthodontic treatment?” only statistically significant differences are found in openness (*F* (2, 319) = 3.80; *p* = 0.023; *ɳ*^2^ = 0.023). The participants who would not undergo any orthodontic treatment showed significantly higher values (*M* = 29.58; DP = 5.34) than the participants who answered “maybe” (*M* = 29.04; DP = 4.95) and “yes” (*M* = 27.66; DP = 4.45).

(c) Differences in the personality traits of the laypeople group, according to the type of orthodontic treatment chosen.

Statistically significant differences were found between the type of orthodontic treatment in relation to neuroticism (*t* (320) = −2.176; *p* = 0.030; *d* = −0.258), openness (*t* (320) = 2.160; *p* = 0.032; *d* = 0.27), and agreeableness (*t* (320) = 3.143; *p* = 0.002; *d* = 0.373). Participants who would prefer to place aligners showed significantly higher values than those who would prefer to place brackets in relation to openness and agreeableness. In the neuroticism dimension, participants who preferred brackets showed significantly higher values than those who preferred aligners (Table 7).

## 4. Discussion

The main objective of our study was to evaluate the relationship between personality traits and the esthetic perception of different orthodontic appliances, considering the psychological and social implications of the smile [20].

In this regard, we began by assessing the esthetic classification of a model's natural smile, the perception of possible orthodontic needs, and the general preference for appliances among laypeople and dentistry workers. The results showed that 72.3% of laypeople gave more positive ratings to the model's smile (beautiful and very beautiful), while the professionals were the least tolerant group, giving less positive ratings (51% as neutral or ugly); these results are to be expected given the specialized training of the professionals. Regarding the improvement of this smile, the results are in line with the evaluation of the smile in which dentistry workers are the group that clearly considers its improvement necessary with orthodontic treatment (43.2% versus 19.3% of laypeople). These findings are consistent with other studies [21–23], which have found that professionals tend to have stronger judgments and needs regarding smile esthetics.

With regard to the sociodemographic variables, and more specifically within gender, we found that with regard to the least positive classification (ugly), there was a predominance of females in the group of dentistry workers compared to males. Satisfaction with dental appearance is known to be correlated with gender [24, 25], with women being more dissatisfied with the appearance of their dentition than men [24]. Several studies have demonstrated this gender difference, identifying women as more concerned about misaligned teeth [26, 27] and with dental conditions, paying more attention to their oral health and are therefore more critical [28, 29] and feel more need for orthodontic treatment than men [26, 27, 30]. This may relate to contemporary media and the prevailing “beauty culture,” which objectify women by maintaining stereotypes regarding the traits that are associated with attractiveness and success [31]. It was hypothesized that this is exacerbated when the background training is higher. It should be noted that 45.9% of the female dentistry group would choose an orthodontic treatment to improve their smile.

Regarding the esthetic preferences in the choice of orthodontic treatment that emerged in our study, there is an agreement between the choices made by the two groups: aligners, followed by esthetic brackets with esthetic wire, esthetic brackets with metal wire, metal brackets with esthetic wire, and metal brackets with metal wire. The attractiveness of appliances decreases with the amount of visible metal, similar to the findings of Ziuchkovski et al. [32] and Rosvall et al. [33], who found lingual appliances and aligners to be the most attractive, followed by ceramic and metal appliances. The preference for aligners may be associated with a lower visual impact on the smile and, therefore, esthetic perception, considering that the majority of participants in both groups were female. The literature suggests that removable orthodontic treatment as having less impact on daily life than fixed orthodontics [34], identifying Invisalign® treatment as being associated with greater satisfaction, better oral health-related quality of life, and less negative impacts on oral health than fixed orthodontics [16, 35–37]. Therefore, in recent years, the presence of metal components has negatively influenced the predisposition and esthetic self-perception to the extent that many people declared themselves willing to invest twice the price to have something esthetic because the smile played a dominant role [38, 39].

Regarding orthodontic treatment with braces, the results suggest that the most important factor in the decision process was the type of bracket rather than the wire used, as esthetic brackets (with and without metal wire) were preferred to metal brackets. These results are consistent with those observed by Ziuchkovski et al. [32], where the appearance of the wire is irrelevant when a stainless steel appliance is used.

In order to evaluate the relationship between personality traits, smile evaluation, and orthodontic appliances in the laypeople group, the NEO-FFI test was used since it offers a complete evaluation of the five dimensions of personality [14, 15, 40, 41].

The results show that people who rated their smile more positively (very beautiful) had high levels of extraversion, openness, and agreeableness and that people with high levels of openness were the only ones who would not change their smile. Regarding the preference for orthodontic treatment, high levels of neuroticism are associated with a greater preference for fixed appliances as opposed to high levels of openness and agreeableness, whose preference is for aligners.

Our results are consistent with the literature indicating that higher personality trait scores of extraversion and openness are associated with a lower impact of orthodontic treatment needs on oral health-related quality of life [42].

Extroverted people are more sociable, friendly, positive, optimistic, affectionate, and cheerful [40], and they have a more positive view of life. As expressed in our results, people with openness higher scores would not improve the smile with an orthodontic treatment, and if they did do it, they would choose aligners. Openness is defined by greater esthetic sensitivity, openness to ideas/intellectual curiosity, range of feelings, and independent judgment. Individuals with high levels in this dimension enjoy the experience, tolerate, and explore the unfamiliar, and are more curious [40], which, combined with esthetic sensitivity, justifies the preference for aligners over conventional metal treatment. These findings are consistent with the study developed by Montero et al. [43], which found a positive association between high levels of extraversion and openness and positive health evaluations.

Agreeableness is an important predictor of social outcomes, and those who score high on this trait are characterized by pro-social, cooperative, and altruistic behavior and the use of emotion-focused coping strategies to seek social support [44]. High agreeableness scores are shown in more ratings of the smile as “very beautiful” and are also associated with the preference for aligners in the case of orthodontic treatment. Agreeable people are likely to seek acceptance from others; therefore, a greater degree of self-perceived malocclusion can result in a much greater psychosocial impact. In addition, orthodontic treatment with aligners has been associated with greater satisfaction, better oral health-related quality of life, and fewer negative oral health outcomes than fixed orthodontics [35, 36, 45, 46].

People with high levels of neuroticism are generally more worried, nervous, hypochondriac, emotionally insecure, with feelings of incompetence, impulsive, and have difficulty coping with stress [40]. In addition, they are more critical, rating the lack of facial symmetry negatively [43], which justifies the stricter rating of the smile in this study (“beautiful”). In this context, their preference for fixed appliances over aligners may reflect their personality traits, as aligner treatment requires self-management and accountability, which can be stressful. These results are in line with other studies that have shown a negative relationship between high levels of neuroticism and the adoption of behaviors that underpin success, such as rigor, persistence, discipline, and, consequently, experience [47, 48]. Feelings of incompetence and impulsiveness may lead them to blame the dentist for the execution, development, and final results of orthodontic treatment.

## 5. Directions for Further Research

This study investigated the relationship between personality traits and orthodontic appliance choices in a nonclinical population based on a hypothetical situation. In this context, it would be relevant to carry out a similar study with a clinical population in order to assess the influence of personality traits on orthodontic appliance choices in a real-life situation. To this end, it would be important to assess pretreatment personality traits and longitudinally evaluate their influence not only on decision-making regarding the type of orthodontic treatment but also on adherence to treatment and the results obtained, especially in terms of satisfaction with the treatment.

It would be important to consider racial backgrounds and cultural and social factors.

## 6. Conclusions

Laypeople tend to devalue the esthetic particularities of the smile, unlike professionals regarding the preferences and grading of the model's smile. Esthetic perception and the need for intervention increase as expertise increases.

The ranking of esthetic preferences that emerged follows that found in the literature: aligners, followed by esthetic brackets, followed by metal brackets, i.e., the attractiveness of the appliance decreases as the amount of visible metal increases.

Esthetic perception is associated with high traits of agreeableness and openness. Neuroticism is most associated with orthodontic treatment with fixed appliances, which are the most conventional.

The results obtained point to the importance of personality characteristics in oral esthetic perception and the consequent decision whether or not to undergo orthodontic treatment and the type of appliance to be used, and may also influence expectations of results and satisfaction, which is an important aspect in clinical practice.

## Figures and Tables

**Table 1:** Gender, education, and age of the groups.

Sociodemographic variables	Total (%)	Laypeople (%)	Dentistry workers (%)	*χ* ^2^	*p*	*Φ*
Gender	461 (100.0)	322 (69.8)	139 (30.2)	0.245	0.620	−0.02
Female	368 (79.8)	259 (80.4)	109 (78.4)	—	—	—
Male	93 (20.2)	63 (19.6)	30 (21.6)	—	—	—
Education						
A level or less	197 (42.7)	139 (43.2)	58 (41.7)	2.83	0.419	0.08
Master's degree or bachelor	199 (43.2)	140 43.5)	59 (42.4)	—	—	—
Specialized MSc	56 (12.1)	39 (12.1)	17 (12.2)	—	—	—
Doctor degree	9 (2.0)	4 (1.2)	5 (3.6)	—	—	—
Age						
18–29	235 (51.0)	184 (57.1)	51 (36.7)	23.518	<0.001	0.23
30–39	67 (14.5)	36 (11.2)	31 (22.3)	—	—	—
40–49	89 (19.3)	63 (19.6)	26 (18.7)	—	—	—
50–59	56 (12.1)	30 (9.3)	26 (18.7)	*—*	*—*	*—*
60–71	14 (3.0)	9 (2.8)	5 (3.6)	*—*	*—*	*—*

% = percentage; *χ*^2^ = Qui-squared; *p* = *p*-value; *Φ* = phi.

**Table 2:** Comparison between laypeople and dentistry workers regarding smile evaluation.

Smile avaluation	Total	Laypeople (%)	Dentistry workers (%)	*χ* ^2^	*p*	*Φ*
How do you rate this smile? (Figure 1)						
Ugly	14 (3.0)	2 (0.6)	12 (8.6)	38.330	<0.001	0.29
Neutral	146 (31.7)	87 (27.0)	59 (42.4)	—	—	—
Beautiful	266 (57.7)	202 (62.7)	64 (46.0)	—	—	—
Very beautiful	35 (7.6)	31 (9.6)	4 (2.9)	—	—	—
If there were your smile, would you improve it with an orthodontic treatment? (Figure 1)						
Yes	122 (26.5)	62 (19.3)	60 (43.2)	—	—	—
No	224 (48.6)	179 (55.6)	45 (32.4)	31.763	<0.001	0.26
Maybe	115 (24.9)	81 (25.2)	34 (24.5)	—	—	—

% = percentage; *χ*^2^ = Qui-squared; *p* = *p*-value; *Φ* = phi.

**Table 3:** Evaluation of the smile according to gender in the group of laypeople and dentistry workers.

Laypeople	Total	Males (%)	Females (%)	*χ* ^2^	*p*	*Φ*
How do you rate this smile? (Figure 1)						
Ugly	2 (0.6)	1 (1.6)	1 (0.4)	5.38	0.146	0.129
Neutral	87 (27.0)	18 (28.6)	69 (26.6)	—	—	—
Beautiful	202 (62.8)	34 (54.0)	168 (64.9)	—	—	—
Very beautiful	31 (9.6)	10 (15.9)	21 (8.1)	—	—	—
If there were your smile, would you improve it with an orthodontic treatment? (Figure 1)						
No	179 (55.6)	42 (66.7)	137 (52.9)	4.69	0.096	0.121
Maybe	81 (25.2)	14 (22.2)	67 (25.9)	—	—	—
Yes	62 (19.2)	7 (11.1)	55 (21.2)	—	—	—

Dentistry workers	Total	Males (%)	Females (%)	*χ* ^2^	*p*	*Φ*

How do you rate this smile? (Figure 1)						
Ugly	12 (8.6)	1 (3.3)	11 (10.1)	3.16	0.367	0.151
Neutral	59 (42.4)	13 (43.3)	14 (42.2)	—	—	—
Beautiful	64 (46.0)	14 (46.7)	50 (45.9)	—	—	—
Very beautiful	4 (2.9)	2 (6.7)	2 (1.8)	—	—	—
If there were your smile, would you improve it with an orthodontic treatment? (Figure 1)						
No	45 (32.4)	15 (50.0)	30 (27.5)	5.48	0.065	0.199
Maybe	34 (24.5)	5 (16.7)	29 (26.6)	—	—	—
Yes	60 (43.2)	10 (33.3)	50 (45.9)	—	—	—

% = percentage; *χ*^2^ = Qui-squared; *p* = *p*-value; *Φ* = phi.

**Table 4:** Frequencies of laypeople rankings.

Types of orthodontic treatment	1st order		2nd order		3rd order		4th order		5th order	
	*n*	(%)	*n*	(%)	*n*	(%)	*n*	(%)	*n*	(%)
MB–MW	51	15.8	51	15.8	37	11.5	52	16.1	131	40.7
MB–AW	10	3.1	44	13.7	66	20.5	137	42.5	65	20.2
AB–MW	9	2.8	39	12.1	149	46.3	61	18.9	64	19.9
AL	216	67.1	55	17.1	18	5.6	14	4.3	19	5.9
AB–AW	36	11.2	133	41.3	52	16.1	58	18.0	43	13.4

*n* = frequencies; % = percentage.

**Table 5:** Frequencies of dentistry workers rankings.

Types of orthodontic treatment	1st order		2nd order		3rd order		4th order		5th order	
	*n*	(%)	*n*	(%)	*n*	(%)	*n*	(%)	*n*	(%)
MB–MW	21	15.1	24	17.3	19	13.7	23	16.5	52	37.4
MB–AW	3	2.2	17	12.2	23	16.5	56	40.3	40	28.8
AB–MW	7	5.0	18	12.9	63	45.3	35	25.2	16	11.5
AL	97	69.8	22	15.8	8	5.8	8	5.8	4	2.9
AB–AW	11	7.9	58	41.7	26	18.7	17	12.2	27	19.4

*n* = frequencies; % = percentage.

**Table 6:** Differences in the personality traits according to the smile evaluation by laypeople in Figure 1.

Personality traits	Smile evaluation	*N*	Mean	Standard deviation	*F*	*df*	*p*	*ɳ* ^2^
Neuroticism	Ugly	2	31.50	0.71	0.84	3 318	0.472	0.008
	Neutral	87	25.38	8.02				
	Beautiful	202	24.35	8.69				
	Very beautiful	31	23.71	9.36				

Extraversion	Ugly	2	19.00	4.24	4.85	3 318	0.003	0.044
	Neutral	87	29.62	5.68				
	Beautiful	202	29.95	5.69				
	Very beautiful	31	32.65	5.77				

Openness	Ugly	2	26.00	1.41	3.90	3 318	0.009	0.035
	Neutral	87	28.39	5.05				
	Beautiful	202	29.08	4.90				
	Very beautiful	31	31.84	5.79				

Agreeableness	Ugly	2	23.50	3.54	3.03	3 318	0.03	0.028
	Neutral	87	33.21	4.14				
	Beautiful	202	33.44	4.69				
	Very beautiful	31	33.52	5.83				

Conscientiousness	Ugly	2	32.50	12.02	0.45	3 318	0.719	0.004
	Neutral	87	34.91	5.78				
	Beautiful	202	34.86	5.81				
	Very beautiful	31	36.00	7.05				

*N =* frequencies*; F =* ANOVA*; df =* degrees of freedom*; p =* significance; *ɳ*^2^ size effect.

**Table 7:** Differences in the personality traits of the laypeople group, according to the type of orthodontic treatment chosen.

Personality traits	Orthodontic treatment	*N*	Mean	Standard deviation	*t*	*df*	*p*	*d*
Neuroticism	Aligners	216	23.89	8.38	−2.176	320	0.030	−0.258
	Brackets	106	26.08	8.77				

Extraversion	Aligners	216	30.25	5.88	0.885	320	0.377	0.105
	Brackets	106	29.64	5.62				

Openness	Aligners	216	29.57	5.12	2.160	320	0.032	0.256
	Brackets	106	28.27	4.94				

Agreeableness	Aligners	216	33.89	4.60	3.143	320	0.002	0.373
	Brackets	106	32.16	4.74				

Conscientiousness	Aligners	216	35.06	6.09	0.393	320	0.695	0.047
	Brackets	106	34.78	5.67				

*N* = frequencies; *t* = *t*-test; *df* = degrees of freedom; *p* = significance; *d* = Cohen test, effect size.

## Data Availability

The authors declare that the data supporting the findings of this study are available within the article.

## Additional Points

*Research Limitations*. This study is innovative, considering that there are few studies on personality and orthodontic appliances in healthy people, making it difficult to compare the results. The method used (online survey and snowball) did not allow for the homogenization of the sample into groups. The sample under study is not representative of the Portuguese population, which means that the results cannot be generalized.

## Supplementary Materials

Supplementary Materials Questionnaire S1: Evaluation of the esthetic perception of orthodontic appliances—a survey questionnaire and the photographic model.
