# Supplementary material for: The Relationship between Personality Profiles and the Esthetic Perception of Orthodontic Appliances
Source: Int J Dent. 2024 Apr 23;2024:8827652. doi: 10.1155/2024/8827652 (PMC11074833; doi:10.1155/2024/8827652)
Supplement: Supplementary Materials — Questionnaire S1: Evaluation of the esthetic perception of orthodontic appliances—a survey questionnaire and the photographic model. [file 8827652.f1.docx]

**Evaluation of the aesthetic perception of Orthodontic Appliances**

This survey “Evaluation of the aesthetic perception of Orthodontic Appliances” is included in an IUCS-CESPU research project coordinated by Prof. Dr. Teresa Pinho, with the aim of better understanding the importance of the aesthetic impact of orthodontics appliances, as well as the factors that can influenced it.

Participation is voluntary and anonymous.

We count on your cooperation.

We thank you in advance for your participation and interest.

The research team:

Professor Dr. Teresa Pinho

The collected data will be processed for scientific research purposes.

**Consent for scientific research purposes**

I agree to voluntarily participate in the survey; I declare that I have been informed about the objectives and confidentiality of this survey, as well as its use for scientific research purposes.

Choose only one of the following options:

- I agree
- I don’t agree

**Generalities**

Choose only one of the following options:

Age:

18 – 29

30 – 39

40 – 49

50 – 59

60 – 71

Gender:

Male

Female

Other

Nationality

Portuguese

Italian

Other

Qualification

A level or less

Master’s Degree or Bachelor

Specialized MSc

Doctor Degree

Study Area

Dentistry and Dental Prosthetics

Dental Hygiene student of other health areas

Engineering

Humanitarian Subjetcs

Social and Economics matters

Arts and Cultural Heritage area

Other

Year of the Degree Course

1^st^ year

2^nd^ year

3^rd^ year

4^th^ year

5^th^ year

6^th^ year

How long have you been practicing orthodontics?

≤ 5 years

≥ 6 and < 10 years

≥ 11 and < 20 years

≥ 20 years

**Aesthetic Perception**

Below it will be present some orthodontic appliances asking you to select from the options presented which one is your favorite

How do you rate this smile?


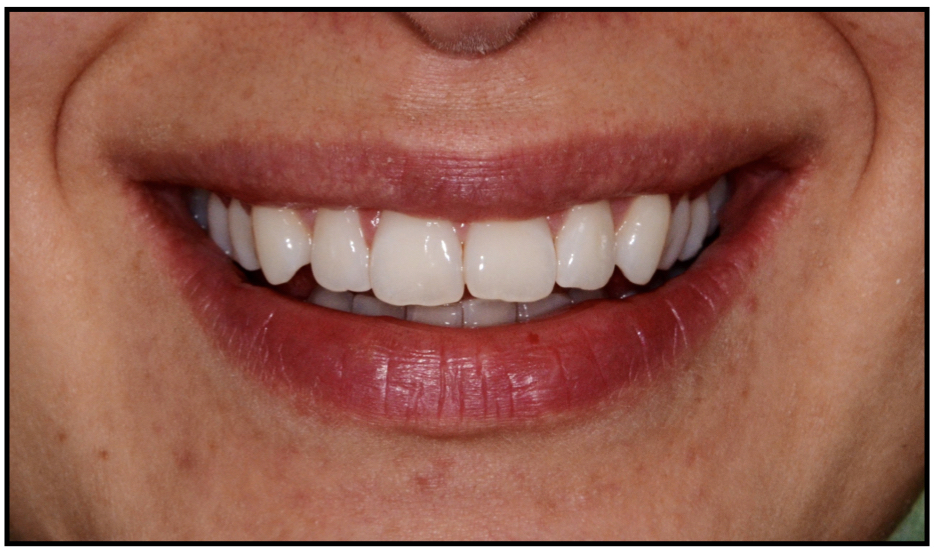


Choose only one of the following:

1 Very bad

2 Ugly

3 Neutral

4 Beautiful

5 Very beautiful

If this were your smile, would you consider it important to improve it with orthodontic treatment? Choose only one of the following options:

Yes

No

Maybe

If you undergo to a treatment, which orthodontic appliance would you like to use?

Choose only one of the following options:

**
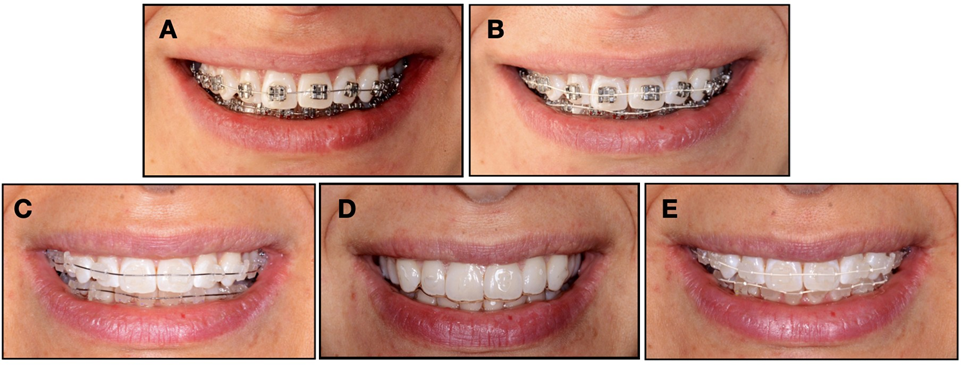
**

A (Fixed Metallic Appliance)

B ( Fixed Metallic Brackets + Aesthetic Wire)

C (Fixed Aesthetic Brackets + Metallic Wire)

D (Aligners)

E (Fixed Aesthetic Brackets + Aesthetic Wire)

Sort the following photos according to your preferences, placing your favorite at the top and the least favorite at the bottom.

**
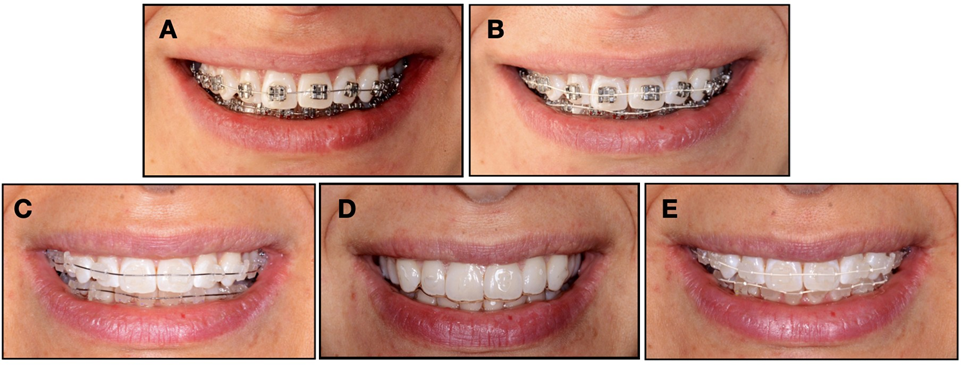
**

Double click or drag and drop.

All answers must be different and ranked in order

A

B

C

D

E

Indicate your preference

**
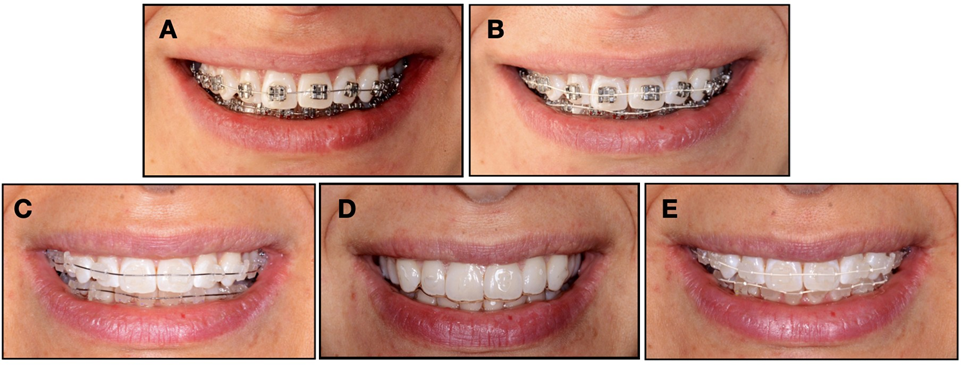
**

A. (Choose only one option)

B

**
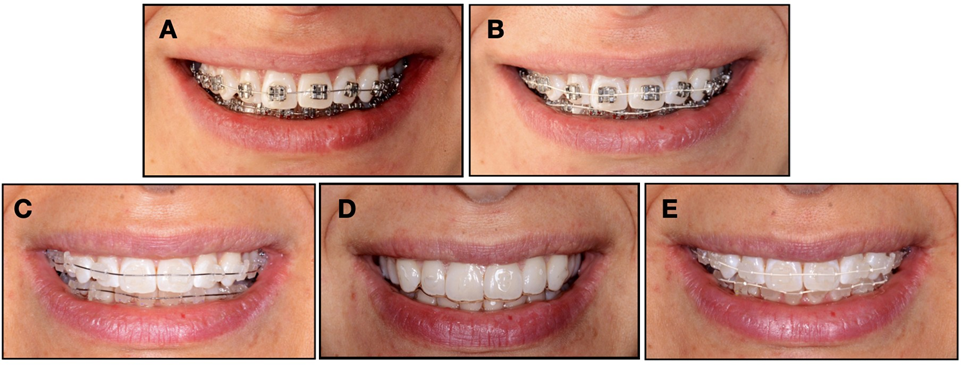

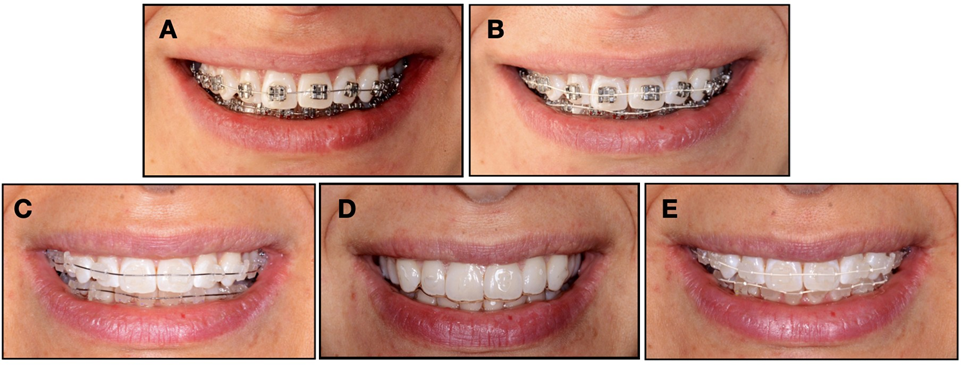
**

A (Choose only one option)

C

**
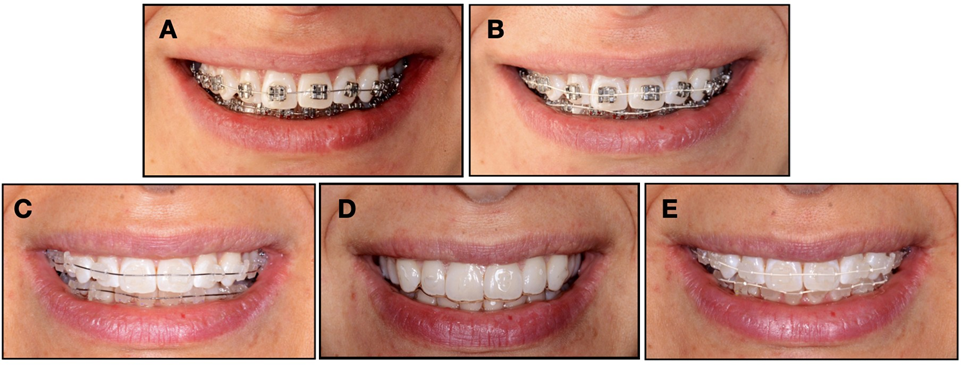

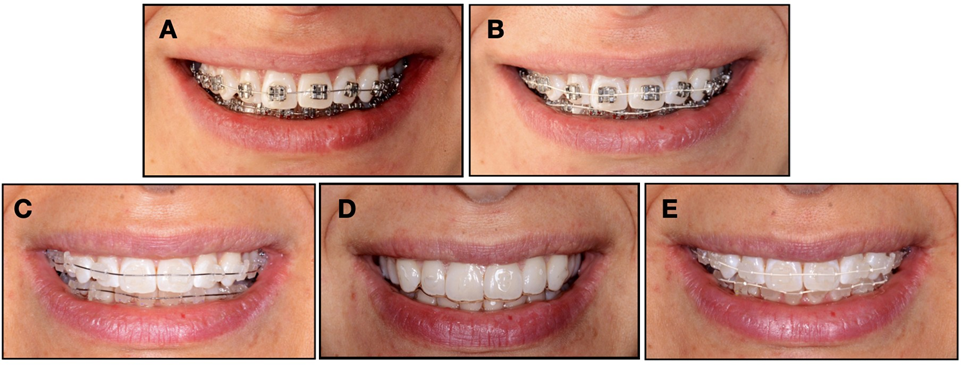
**

E (Choose only one option)

C

**
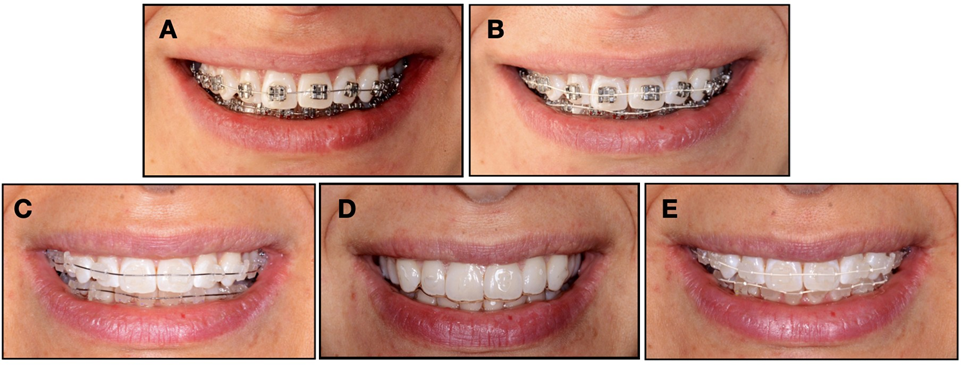

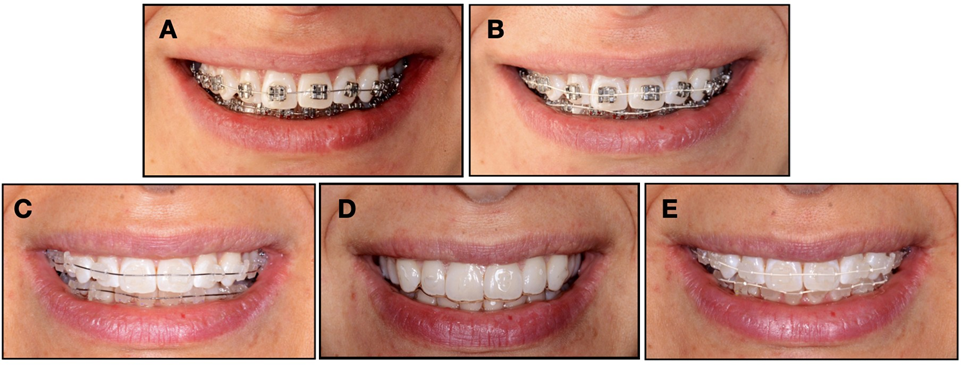
**

B (Choose only one option)

E

**
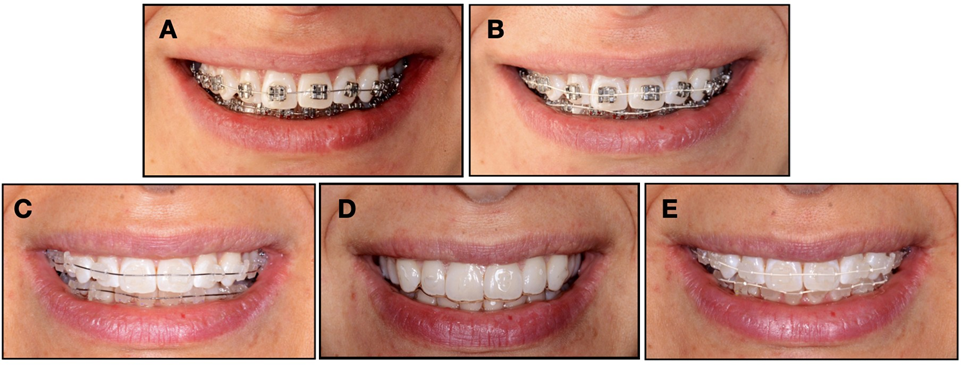
** **
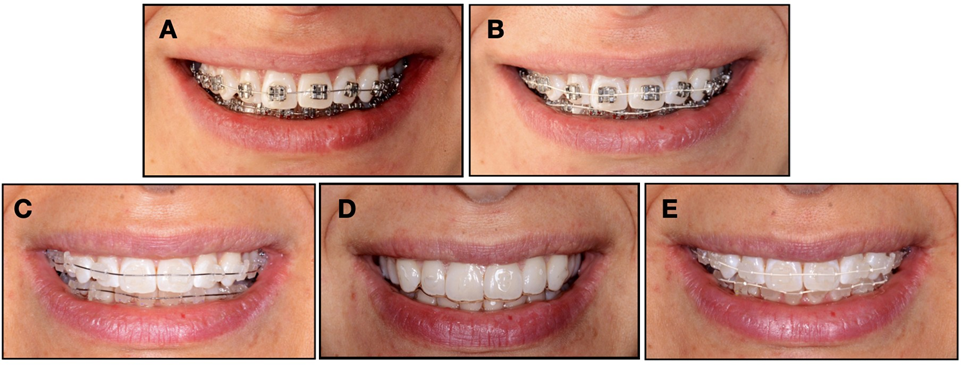
**

D (Choose only one option)

E

Have you already undergone any orthodontic treatment?

Never

Yes, right now

Yes, but more than 2 years ago

Yes, but more than 5 years ago

What type of orthodontic appliance do you use / have you used?

(Choose only one option)

Fixed Metallic

Fixed Aesthetic

Aligners

Do the results obtained coincide with those hoped for?

Yes

No

More or less

**Personality profiles**

Read each of the following statements carefully and tick the one that best represents your opinion. Answer all the questions.

|  | Strongly disagree | Disagree | Neither agree nor disagree | Agree | Strongly Agree |
| --- | --- | --- | --- | --- | --- |
| I am not a worrier |  |  |  |  |  |
| I like to have a lot of people around me |  |  |  |  |  |
| I don’t like to waste my time daydreaming |  |  |  |  |  |
| I try to be courteous to everyone I meet |  |  |  |  |  |
| I keep my belongings neat and clean |  |  |  |  |  |
| I often feel inferior to others |  |  |  |  |  |
| I laugh easily |  |  |  |  |  |
| Once I find the right way to do something. I stick to it |  |  |  |  |  |
| I often get into arguments with my family and co-workers |  |  |  |  |  |
| I’m pretty good about pacing myself so as to get things done on time |  |  |  |  |  |
| When I’m under a great deal of stress, sometimes I feel like I’m going to pieces |  |  |  |  |  |
| I don’t consider myself especially “light hearted” |  |  |  |  |  |
| I am intrigued by the patterns I find in art and nature |  |  |  |  |  |
| Some people think I’m selfish and egotistical |  |  |  |  |  |
| I am not a very methodical person |  |  |  |  |  |
| I rarely feel lonely or blue |  |  |  |  |  |
| I really enjoy talking to people |  |  |  |  |  |
| I believe letting students hear controversial speakers can only confuse and mislead them |  |  |  |  |  |
| I would rather cooperate with others than compete with them |  |  |  |  |  |
| I try to perform all the tasks assigned to me conscientiously |  |  |  |  |  |
| O often feel tense and jittery |  |  |  |  |  |
| I like to be where the action is |  |  |  |  |  |
| Poetry has little or no effect on me |  |  |  |  |  |
| I tend to be cynical and skeptical of others’ intentions |  |  |  |  |  |
| I have a clear set of goals and work toward them in an orderly fashion |  |  |  |  |  |
| Sometimes I feel completely worthless |  |  |  |  |  |
| I usually prefer to do things alone |  |  |  |  |  |
| I often try new and foreign foods |  |  |  |  |  |
| I believe that most people will take advantage of you if you let them |  |  |  |  |  |
| I waste a lot of time before setting down to work |  |  |  |  |  |
| I rarely feel fearful or anxious |  |  |  |  |  |
| I often feel as if I’m bursting with energy |  |  |  |  |  |
| I seldom notice the moods or feelings that different environments produce |  |  |  |  |  |
| Most people I know like me |  |  |  |  |  |
| I work hard to accomplish my goals |  |  |  |  |  |
| I often get angry at the way people treat me |  |  |  |  |  |
| I am a cheerful, high-spirited person |  |  |  |  |  |
| I believe we should look to our religious authorities for decisions on moral issues |  |  |  |  |  |
| Some people think of me as cold and calculating |  |  |  |  |  |
| When I make a comment, I can always be counted on to follow through |  |  |  |  |  |
| Too often when things go wrong, I get discouraged and feel like giving up |  |  |  |  |  |
| I am not a cheerful optimist |  |  |  |  |  |
| Sometimes when I am reading poetry or looking at a work of art, I feel a chill or wave of excitement |  |  |  |  |  |
| I’m hard-headed and tough-minded in my attitudes |  |  |  |  |  |
| Sometimes I’m not as dependable or reliable as I should be |  |  |  |  |  |
| I am seldom sad or depressed |  |  |  |  |  |
| My life is fast-paced |  |  |  |  |  |
| I have little interest in speculating on the nature of the universe or the human condition |  |  |  |  |  |
| I generally try to be thoughtful and considerate |  |  |  |  |  |
| I am a productive person who always gets the job done |  |  |  |  |  |
| I often feel helpless and want someone else to solve my problems |  |  |  |  |  |
| I am a very active person |  |  |  |  |  |
| I have a lot of intellectual curiosity |  |  |  |  |  |
| If I don’t like people, I let them know it |  |  |  |  |  |
| I never seem to be able to get organized |  |  |  |  |  |
| At times I have been so ashamed I just wanted to hide |  |  |  |  |  |
| I would rather go my own way than be a leader of others |  |  |  |  |  |
| I often enjoy playing with theories or abstract ideas |  |  |  |  |  |
| If necessary, I am willing to manipulate people to get what I want |  |  |  |  |  |
| I strive for excellence in everything I do |  |  |  |  |  |
